# The indirect and direct pathways between physical fitness and academic achievement on commencement in post-compulsory education in a historical cohort of Danish school youth

**DOI:** 10.1186/s12889-017-4712-y

**Published:** 2017-09-11

**Authors:** Mikkel Porsborg Andersen, Liis Starkof, Maurizio Sessa, Rikke Nørmark Mortensen, Henrik Vardinghus-Nielsen, Henrik Bøggild, Theis Lange, Christian Torp-Pedersen

**Affiliations:** 1 Public Health and Epidemiology Group, Department of Health Science and Technology, Aalborg University, Niels Jernes Vej 12 Øst, 9220 Aalborg, Denmark; 2 Section of Biostatistics, Department of Public Health, Faculty of Health and Medical Sciences, University of Copenhagen, Øster Farimagsgade 5, 1353 København K, Denmark; 3 Department of Experimental Medicine, Section of Pharmacology “L. Donatelli”, Second University of Naples, Via De Crecchio 7, 80138 Naples, Italy; 4 Department of Clinical Epidemiology, Aalborg University Hospital, Sdr. Skovvej 15, 9000 Aalborg, Denmark; 5 Center for Statistical Science, Peking University, Beijing, China

**Keywords:** Physical fitness, Academic achievement, Post-compulsory education, Scholastic abilities, Causal inference, Mediation analysis

## Abstract

**Background:**

Some studies have found positive associations between physical fitness and academic achievements. Pupils’ academic achievements should indicate scholastic abilities to commence a post-compulsory education. However, the effect magnitude of physical fitness and academic achievements on commencement in post-compulsory education is unknown. We examined the pathways between physical fitness and academic achievement on pupils’ commencement in post-compulsory education.

**Methods:**

This historical cohort study followed 530 girls and 554 boys from the Danish municipality of Aalborg in the period 2008–2014, 13 to 15 years old in 2010. Physical fitness was assessed through a watt-max cycle ergometer test represented as VO_2_max (mL·kg^−1^·min^−1^). Academic achievement, commencement status and information on covariates were obtained from Danish nationwide registers. Causal inference based mediation analysis was used to investigate the indirect and direct pathways by separating the total effect of physical fitness on post-compulsory education commencement.

**Results:**

Adjusting for sex, age, ethnicity and socioeconomic status, the overall mediation analysis showed an odds ratio (OR) of 1.87 (95% confidence interval (CI): 1.30; 2.73) for the total effect, corresponding to an increase in odds of post-compulsory education commencement when the physical fitness was increased by 10 units of VO_2_max. The separated total effect showed a natural direct OR of 1.36 (95% CI: 0.93; 1.98) and a natural indirect (i.e., through academic achievement) OR of 1.37 (95% CI: 1.20; 1.57). Thus, 51% (95% CI: 27%; 122%) of the effect of physical fitness on post-compulsory education commencement was mediated through academic achievement.

**Conclusion:**

Physical fitness had a positive effect on post-compulsory education commencement. A substantial part of this effect was mediated through academic achievement.

## Background

Education is a key element for human development and economic growth [1, 2], associated with better health status, work skills, and lower crime rates in society [3]. In the last decade, some countries have focused on reforms to improve educational attendance [1, 4, 5] to achieve the level of knowledge and educational attainment the workplaces require [1]. In England, legislation has been applied in an attempt to improve preparation and persistence towards commencement of post-compulsory education by extending the length of pupils’ mandatory education [4]. Similarly, in 2014, Danish policy makers decided to introduce a school legislation that prioritizes an extended school day with additional mandatory time for physical activity as a way to improve health, maintain motivation and support academic subjects [5]. Despite policy makers’ willingness to explore new approaches to improve attendance in post-compulsory education, it is unknown whether physical activity influences attendance. The intensity, duration, and frequency of all physical activities and exercise a person performs determine the physical fitness level [6, 7]. Research has found inconsistent result in linking physical activity to academic achievements [8, 9]. Further, previous studies have found a positive association between physical fitness and academic achievements in pupils, suggesting that improving physical fitness could have a positive effect on their academic achievements [9–17]. This indicates that a way, in which physical activity may affect academic achievements, is through physical fitness as partially supported by Lambourne et al. [18]. Studies have likewise found that improving the physical fitness level have a positive impact on multiple neurological functions [19–24] that have been associated with better learning ability [9, 19, 20, 22, 23, 25]. This suggests that the positive impacts on academic achievements might be a result of improved physical fitness level. To the authors’ knowledge, no studies have yet investigated whether physical fitness could affect pupils’ commencement into post-compulsory education. Post-compulsory education is defined as all upper secondary and vocational education and training programs equivalent to level 3 in the International Standard Classification of Education (ISCED) [26, 27]. A factor that could influence pupils’ commencement is academic achievements from compulsory education, because this should represent pupils’ scholastic abilities and indicate their ability to continue into a post-compulsory education. This is supported by a report produced by the Danish National Centre for Social Research, illustrating that the more pupils at a school who obtain a grade point average above seven on the Danish seven-point grading scale (e.g., equivalent to a C on the European Credit Transfer and Accumulation System (ECTS) [28]), the greater the proportion of pupils who will commence a post-compulsory education [29]. This suggests that academic achievements could potentially be an intermediate on the pathway between pupils’ physical fitness and attendance in post-compulsory education. The objective of this study is to examine the pathways between physical fitness and academic achievement on post-compulsory education commencement; the pupils’ academic achievement from compulsory education are treated as a mediator in this relation.

## Methods

### Data source

All data for this cohort study were obtained from a health examination conducted by the Danish municipality of Aalborg in 2010 and from nationwide registers. These data were linked through a unique ten digit civil registration number, which all Danish residents are assigned at birth or when establishing residence in Denmark. Through this number, it is possible to connect pupils to their parents or guardians and link individual data from different registers [30]. The identities of parents or guardians, as well as age, gender, ethnicity of all pupils were obtained from the Danish Civil Registration System [31]. The municipality of Aalborg granted permission, to place the health examination data from 2008 and 2010 in the research environment of Statistics Denmark with the approval of the Danish Data Protection Agency (Journal number: 2014–41-2842). The pupils’ physical fitness levels were measured in a health examination in 2010. The pupils’ grade levels at graduation from compulsory education, academic achievements and commencement or deselection of post-compulsory education were obtained from two registers: the Student Register and the Academic Achievement Register [32]. Pupils’ hospital contacts were obtained from the Danish National Patient Register [33]. Information on parental income, education and civil status was obtained from the Income Register, the Population Educational Register and the Danish Civil Registration System, respectively [31, 32, 34]. All the individual information was organized and stored in the research environment of Statistics Denmark. In Denmark register-based studies that are anonymized do not require informed consent or ethical approval.

### Participants

In 2008, the Danish municipality of Aalborg invited all public elementary schools with ninth grade class levels to participate in health examinations of their sixth-grade pupils. The health examinations were repeated when the pupils attended eighth grade in 2010. In total, 1544 sixth-grade pupils out of 1638 participated in the health examinations. A total of 1164 pupils had their physical fitness re-measured in eighth grade in 2010, during which 1771 pupils were enrolled in eighth grade in the municipality public elementary schools. However, because of the original study design, the pupils were required to have participated in the first examination to be included in the second health examination. To minimize the timespan between the pupils’ physical fitness and academic achievements measurements, the data from the eighth-grade health examination in 2010 were used to represent the pupils’ physical fitness. Of the 1164 pupils, 80 were excluded for the following reasons: personal identification number was registered incorrectly (*n* = 3); parental data could not be linked to the pupil (*n* = 10); no information was available regarding parental education and income, for at least one of the parents (*n* = 4); could not be linked to the Academic Achievement Register (*n* = 23); and missing data in one or more of the mandatory exams (*n* = 40). In total, 1084 pupils without missing data for any of the variables were included in this study. The gender distribution was 530 (48.9%) girls and 554 (51.1%) boys with an age range between 13 and 15 years in 2010.

### Physical fitness

In the literature, physical fitness is defined as a set of components that include cardiorespiratory endurance, muscular endurance, muscular strength, flexibility and body composition [7, 15]. In this study, only cardiorespiratory endurance was used to represent pupils’ physical fitness. The pupils’ cardiorespiratory endurance level was measured through a watt-max test conducted on a Monark 874E cycle ergometer under observation by test instructors during the health examinations in 2010. The pupils began the test with a workload of 50 or 75 watts, depending on the pupil’s individual body composition, and cycled with a cadence of 50 rpm. Every third minute, the workload was increased by 25 watts until the threshold of fatigue was reached. The result from this test was calculated as watt-max = W_1_ + (W_2_·T_1_/180), in which W_1_ represents the workload in watts at the last fully completed section, and W_2_ and T_1_ are the increase in watts and the number of seconds in the last not fully completed section, respectively. The result from the watt-max calculation is used to obtain the pupils’ relative maximal oxygen consumption (VO_2_max; mL·kg^−1^·min^−1^), which has been found to be representative of the direct measurement of VO_2_max [35, 36]. The VO_2_max measurements provide a continuous value used to represent, the physical fitness level of each pupil.

### Academic achievement

All Danish elementary schools organize obligatory exams for ninth-grade pupils. These exams are constructed by the Danish Ministry of Education and indicate the completion of compulsory education at the end of the ninth grade [37]. Pupils attend nine mandatory exams including writing, reading, spelling, oral and structured Danish, oral English, science, and oral and written mathematics. The assessment follows the Danish seven-point scale, which assigns the following numeric values: 12, 10, 7, 4, 02, 00, and −3, corresponding to the letters A, B, C, D, E, Fx, and F from the ECTS, respectively [28]. No official conversion between ECTS- and United States grading scales exist, however an unauthorized conversion have been described elsewhere [14]. All pupils have the possibility to voluntarily attend an additional year of compulsory education at the tenth grade, to improve their academics and prepare them for post-compulsory education [37]. Pupils choosing this possibility have mandatory exams at the end of the tenth grade, conducted and graded equally to ninth grade. Pupils in tenth grade can choose between repeating the exams at the ninth grade level or completing the exams at an increased difficulty corresponding to the tenth-grade level [37]. However, in both cases, pupils can improve their academic achievements from the ninth grade exams. In this study, the numeric value of all nine obligatory exams from the ninth grade was averaged to represent the pupil’s individual academic achievement as a grade point average. If pupils attended tenth grade and scored better in exams equivalent to one of the nine obligatory exams, the new exam score was used instead of the old. Pupils were required to have exams scores for all nine mandatory exams to be included in the analysis. In Denmark, all parents or guardians are obliged to ensure that their child receives a minimum of nine years of compulsory education or equivalent schooling [37]. The minimum age for a child to start compulsory education is five years [37], therefore the earliest age a pupil can finish compulsory education is at the age of fourteen.

### Post-compulsory education

In Denmark, a post-compulsory education (equivalent to a youth education) is defined as all upper secondary and vocational education and training programs [26]. These educations or programs are the next possible level of education to attend after finishing compulsory education at either the ninth- or tenth-grade [26] and are defined as level 3 in the International Standard Classification of Education (ISCED) [27]. In an American context, the Danish post-compulsory education would approximately be equivalent to the upper part of secondary- and the lower part of postsecondary education. The post-compulsory education attendance status was evaluated through the Student Register after completion of the compulsory education either at the ninth or tenth grade level. The pupil’s commencement or deselection of post-compulsory education was categorized as a binary outcome, using the latest updated information from the Student Register on September 30, 2014 [38].

### Ethnicity

The ethnicity of the pupils was categorized into ethnic Danes or immigrants/descendants. Ethnic Danes are defined as individuals who have at least one parent with a Danish citizenship and originating from Denmark [39]. Immigrants are defined as individuals born abroad whose parents were born outside of Denmark and have no Danish citizenship [39]. Descendants are defined as individuals born in Denmark whose parents are born abroad and without a Danish citizenship [39].

### Socioeconomic status

Socioeconomic status was represented by parental income and education levels in 2010. The income levels were obtained for both parents using an equivalized income provided by Statistic Denmark, allowing comparisons between households with different numbers of family members [40]. If the parents did not share household, the equivalized income would differ between the parents, and in these cases, the highest income was chosen to represent the income level. The income levels were divided into quartiles. The parents´ education level was categorized into four groups according to the ISCED [27] in which level one (ISCED 0–2) represents early childhood, primary education and lower secondary educations, with a duration of 8–11 years. Level two (ISCED 3) represents general upper secondary education and vocational upper secondary education with a duration between 2 and 5 years. Level three (ISCED 5–6) includes short-cycle tertiary, medium-length tertiary and bachelor’s-level educations or the equivalent with a duration of 2–4 years. Level four (ISCED 7–8) refers to second-cycle, master’s-level or the equivalent and PhD-level educations with a duration of 1–7 years. In Denmark, the ISCED education level 4 is not used [27]. If the parents were categorized into different educational levels, the highest level was chosen to represent the parental education level.

### Overall health

Pupils overall health was assessed through the Danish National Patient Register [33] by evaluating all kinds of hospital contacts that occurred within 365 days prior to measurements physical fitness in 2010. The pupils’ were categorized binary as either been in hospital contact or not been in hospital contact.

### Data analysis

**Fig. 1:**
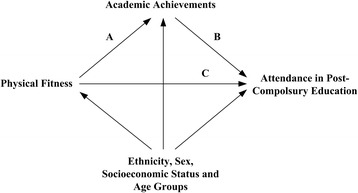
Causal diagram reflecting the study mediation hypothesis. Causal diagram reflecting the study mediation hypothesis. Arrows A + B show the natural indirect effect pathway, and arrow C shows the natural direct effect pathway

Overall differences in the study population and between genders were tested using a t-test, chi-squared tests or Fischer’s exact test when relevant. A significance level of 0.05 was used throughout the analyses. Mediation analysis was used to assess the pathways between physical fitness → academic achievement → commencement in post-compulsory education. Specifically, an imputation-based approach for nested counterfactuals [41, 42] was used. The analysis was adjusted for all covariates previously described (Fig. 1). The approach divides the total effect of physical fitness on post-compulsory education commencement into the natural direct and the natural indirect effect (i.e., the effect mediated through the mediator academic achievement). The natural indirect effect measures the combined effect of physical fitness affecting academic achievement and the subsequent effect of this change in academic achievement on post-compulsory education commencement. The estimation approach used a logistic regression model as the so-called natural effect model [41, 42], leading to odds ratio (OR) estimates for total-, direct-, and indirect effects. The proportion mediated is the ratio between the natural indirect and total effects on a log odds ratio scale. Logistic regression was also used as the imputation model for the outcome given physical fitness, academic achievement and the set of covariates. Bivariate analyses of overall health showed no significant reasons for including this variable in the mediation analyses. The covariates used in both models were sex, age, ethnicity, and socioeconomic status; both models included the main effects and no interaction terms. Special attention was devoted to examining a potential non-linear effect of academic achievement and the interaction between sex and either academic achievement or physical fitness. Bootstrapping with 10,000 bootstrap samples was used to conduct inference on the proportion mediated, and 1000 bootstrap samples were used to compute 95% confidence intervals (CI) for the total-, direct-, and indirect effects. All data management was performed using SAS software version 9.4 (SAS Institute Inc., Cary, NC, USA), and statistical analyses were conducted using the R statistical software package, version 3.2.4 (Development R-core team), particularly the medflex package version 0.6.0.

## Results

### Study population

The population characteristics are described in Table 1. In total, 87 (8.0%) pupils had deselected and 997 (92.0%) had commenced a post-compulsory education by September 30, 2014. In terms of the pupils’ ethnic distributions, 988 (91.1%) were ethnic Danes, and 96 (8.9%) were immigrants/descendants. There was difference between ethnic Danes and immigrants/descendants in physical fitness and academic achievement. On average, ethnic Danes had 2.29 mL·kg^−1^·min^−1^ (95% CI: 0.63; 3.96) higher physical fitness compared to immigrants/descendants. A similar relation was found for academic achievement: ethnic Danes scored on average 0.87 (95% CI: 0.43; 1.31) higher in academic achievement compared to immigrants/descendants. A bivariate analysis of the pupils’ sex revealed differences in both physical fitness and academic achievement. Girls physical fitness was on average 6.56 mL·kg^−1^·min^−1^ (95% CI: -7.39; −5.74) lower than for boys. The opposite relation was observed for academic achievement where girls scored on average 0.79 (95% CI: 0.53; 1.05) grades higher than boys. Bivariate analysis likewise revealed no differences between pupils overall health status in neither physical fitness nor academic achievement. Pupils with no hospital contact had on average 0.22 mL·kg^−1^·min^−1^ (95% CI: -1.01; 1.45) higher physical fitness than pupils with a hospital contact. Pupils with no hospital contact scored on average 0.27 (95% CI: -0.05; 0.60) grades better than pupils with a hospital contact.

**Table 1:** Demographic characteristics of the study participants

Characteristics/Variables	Post-compulsory education		Total (*N* = 1084)	*P*-value
	Deselection (*N* = 87)	Commencement (*N* = 997)		
Age N (%)				
13 years	12 (13.8)	142 (14.2)	154 (14.2)	
14 years	64 (73.6)	794 (79.6)	858 (79.2)	
15 years	11 (12.6)	61 (6.1)	72 (6.6)	0.064
Ethnicity N (%)				
Danes	79 (90.8)	909 (91.2)	988 (91.1)	
Immigrants/Descendants	8 (9.2)	88 (8.8)	96 (8.9)	0.845
Gender N (%)				
Girls	44 (50.6)	486 (48.7)	530 (48.9)	
Boys	43 (49.4)	511 (51.3)	554 (51.1)	0.830
Household income categories N (%)				
Lowest	33 (37.9)	226 (22.7)	259 (23.9)	
Second lowest	30 (34.5)	241 (24.2)	271 (25.0)	
Second highest	14 (16.1)	263 (26.4)	277 (25.6)	
Highest	10 (11.5)	267 (26.8)	277 (25.6)	<0.001
Parental education categories N (%)				
ISCED 0–2	13 (14.9)	60 (6.0)	73 (6.7)	
ISCED 3	52 (59.8)	435 (43.6)	487 (44.9)	
ISCED 5–6	15 (17.2)	334 (33.5)	349 (32.2)	
ISCED 7–8	7 (8.0)	168 (16.9)	175 (16.1)	<0.001
Physical fitness Mean (SD)				
	41.2 (7.8)	45.1 (7.6)	44.8 (7.7)	<0.001
Academic achievement Mean (SD)				
	5.1 (2.1)	7.4 (2.1)	7.2 (2.2)	<0.001
Pupils overall health status N (%)				
No hospital contacts	72 (82.8)	804 (80.6)	876 (80.8)	
Hospital contacts	15 (7.2)	193 (19.4)	208 (19.2)	0.735

Demographic characteristics of the study participants, including age, ethnicity, sex, household income, parental education, physical fitness, academic achievement and pupils’ hospital contacts divided into commencement or deselection of post-compulsory education. The results are based on data from both Danish nationwide registers and a health examination conducted by the Danish municipality of Aalborg, representing a total of 1084 elementary school pupils in the municipality of Aalborg, Denmark.

### Mediation analysis results

**Fig. 2:**
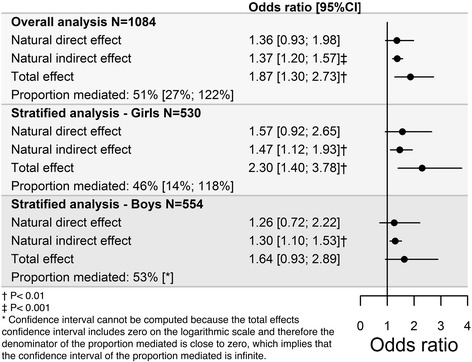
Mediation analysis results. Mediation results for the overall population and stratified by sex, displaying the natural direct and indirect effects as well as the total effect and the proportion mediated between physical fitness, academic achievement and attendance in post-compulsory education, adjusted for age, ethnicity, sex, and socioeconomic status

All results from the mediation analysis are presented in Fig. 2. All total effect OR estimates of the mediation analyses show an increase in odds of commencement in post-compulsory education when physical fitness is increased by 10 units of mL·kg^−1^·min^−1^ (VO_2_max). All natural direct effect OR estimates from the mediation analyses show an increase in odds of commencement in post-compulsory education when the physical fitness level of all pupils is increased by 10 units of VO2max from the same arbitrary reference level without changing their academic achievement. All natural indirect effect OR estimates from the mediation analyses show an increase in odds of commencement in post-compulsory education, when the pupils academic achievement level is changed from what it would be if all pupils had the same arbitrary reference level of physical fitness, to what it would be if all pupils had the physical fitness 10 units above the reference level while actually keeping the physical fitness fixed at the reference level. The overall analysis was adjusted for sex, age, ethnicity and socioeconomic status. From this analysis, an OR of 1.87 (95% CI: 1.30; 2.73) was obtained for the total effect. The natural direct effect component of the total effect represents an OR of 1.36 (95% CI: 0.93; 1.98), while the confidence interval is too wide to allow the existence of other pathways not passing through academic achievement to be ruled out. The natural indirect effect component of the total effect represents an OR of 1.37 (95% CI: 1.20; 1.57), resulting in strong evidence of a natural indirect effect of physical fitness mediated through academic achievement. The proportion of the effect of physical fitness on commencement in post-compulsory education mediated by academic achievement was 51% (95% CI: 27%; 122%), indicating a substantial mediation. An interaction analysis showed no effect modification on either academic achievement or physical fitness by sex, implying no effect differences between girls and boys. However, because of sex differences in both physical fitness and academic achievement a stratified analysis was performed.

Stratifying the overall analysis by sex and adjusting for age, ethnicity and socioeconomic status, the total effect shows an OR of 2.30 (95% CI: 1.40; 3.78) for girls and 1.64 (95% CI: 0.93; 2.89) for boys. The natural direct effect shows an OR of 1.57 (95% CI: 0.92; 2.65) for girls and 1.26 (95% CI: 0.72; 2.22) for boys, showing insufficient evidence of a natural direct effect of physical fitness on post-compulsory education commencement for both sexes. For girls, the natural indirect effect shows an OR of 1.47 (95% CI: 1.12; 1.93) and OR of 1.30 (95% CI: 1.10; 1.53), for boys, resulting in strong evidence of a natural indirect effect of physical fitness mediated through academic achievement, thereby providing evidence of an indirect pathway for both sex. The total proportion mediated is 46% (95% CI: 14%; 118%) for girls and 53% for boys. The boys 95% confidence interval cannot be computed because the total effects confidence interval included zero on the logarithmic scale, indicating that the proportion mediated is infinite.

## Discussion

The main finding of this study was a positive effect of physical fitness on attendance in post-compulsory education. Better physical fitness resulted in an improved probability of post-compulsory education commencement. However, the effect was mediated through the pupils’ academic achievement, indicating that the direction of the causal pathway in the relation may go from physical fitness over academic achievement to post-compulsory education. This study contributes to the literature by revealing an impact of pupils’ physical fitness on post-compulsory education attendance and by establishing the relation between physical fitness, academic achievement and post-compulsory education commencement. An explanation for the relation could be that physical activity and exercise improve physical fitness [6, 7], which might promote the development of neurological functions, brain structure and growth [17, 19–24], all which are crucial for cognition, memory and learning [9, 17, 19, 20, 22, 23, 25]. All these factors may significantly influence pupils’ academic achievements or scholastic abilities, which are supported by Chaddock et al. [22]; therefore, this relation is a possible explanation for pupils’ choice or ability to commence post-compulsory education. Another underlying mechanism that could influence the relation is the pupils’ motivation and self-esteem, which was not possible to investigate in this study. Previous studies suggest that pupils with a high self-esteem or who are motivated regarding academics and physical activity may perform better in both disciplines [10, 13, 43–45]. However, a person’s physical fitness level is a product of the physical activity and exercise performed [6, 7], and improvements in physical activity and exercise can have a positive effect on an individual’s self-esteem [45–47]. Thus, it could be argued that physical fitness level to some extent reflects a pupils’ self-esteem.

This study did not establish a direct effect of physical fitness on attendance in post-compulsory education. However, physical fitness indirectly affected attendance in post-compulsory education through academic achievements, emphasizing a connection between physical fitness and academic achievements. In the overall mediation analysis, the lower bound of the 95% confidence interval for the proportion mediated was 27%, demonstrating a substantial mediation of physical fitness through academic achievements. Since the confidence interval also included 100%, it cannot be excluded that academic achievement might be the only mediator between physical fitness and commencement in post-compulsory education. This study therefore confirms prior findings of an association between physical fitness and academic achievements [11, 13–17, 43] and contributes to the literature by indicating the pathway of this association. The present study found no effect modification by sex in the relation between physical fitness, academic achievement and post-compulsory education commencement. However, this study found sex differences in both academic achievement and physical fitness in accordance with prior findings [48–50]. This study did not establish sex differences in post-compulsory education attendance; nevertheless, the findings regarding sexes showed differences in physical fitness and academic achievement, emphasizing the importance of stratifying analyses when investigating relations between physical fitness, academic achievements and post-compulsory education attendance in youth. The study did likewise not establish differences in physical fitness, academic achievement or commencement in post-compulsory education between pupils who had hospital contact a year prior to the physical fitness measurements compared to pupils not being in contact. This was as expected in a cohort of this age groups as diseases among pupils of this age is rare, also pupils sickness would have been reflected in their performance of the physical fitness measurement. Physical fitness is a strong marker for an individual’s health status, and there are numerous health benefits of having a good fitness level [6, 10, 51–53]. The overall study findings suggest that spending time on physical activity and exercise that improves physical fitness in the school setting could be beneficial for both scholastic abilities and health. The school environment could strive to make the daily setting a place that improves both core academics and health in pupils. Pupils spend much of their waking hours in school, making this an ideal setting to introduce pupils to good and healthy habits such as being physical active in everyday life. Pupils who have a physically active lifestyle in their earlier years of life are more likely to continue this lifestyle throughout adolescence and into adulthood [45, 54, 55]. This can promote general health and delay the onset of unhealthy lifestyle and related diseases [6, 21]. Identifying ways to improve scholastic abilities and education attendance in youth would be beneficial for the individual and for the societal development, in that better-educated populations leads to improved human health, development and economic growth [1–3]. Consequently, it would be interesting to conduct further research in this area to clarify the relations between pupils’ health and scholastic abilities. In particular, a randomized controlled trial that investigated whether an increase in pupils’ physical fitness actually leads to improved academic achievements would be of great novelty for the health and education areas.

### Limitations

The current study results are based on a mediation analysis that assumes no unmeasured confounding [41]. However, in observational study designs, this assumption does not always hold. There could potentially be underlying mechanisms that influence the observed relation. In this study it was not possible to investigate a potential effect of pupils’ social support or maturity state, which may influence the studied relationship. As the pupils’ deselection of post compulsory education is a rare event this could affect the statistical power of the study. However, the width of the confidence intervals reflects this, therefore the authors do not believe that this have had a negative impact on the study. Finally, the used study design could have underestimated the proportions mediated because of the estimation approach used to represent the pupils’ academic achievement. Some of the nine mandatory exams could have a lesser importance for the individual pupil’s choice of post-compulsory education, and the subject could therefore be neglected by the pupil. This could introduce systematic error in the true estimate of the scholastic ability of the pupil. Although this information was impossible to obtain and take into account, the authors do not believe that the error occurred in the current study because the year in which the pupils attended the ninth or tenth grade, no individual exam could have influenced their possibility to commence a post-compulsory education.

### Strengths

To the authors’ knowledge, the present study is the first study to investigate the pathways between physical fitness, academic achievement and commencement of a post-compulsory education; it is also the first study to establish a positive effect of physical fitness mediated through academic achievement on post-compulsory education commencement. The major strengths included the cohort study design, using detailed information on the pupils’ physical fitness levels, the exact background information from administrative nationwide registers and a complete follow-up of the participants. Although the study includes a relatively limited number of pupils, all the public elementary schools in the municipality of Aalborg were included, making the generalizability higher by reflecting all ethnic and socioeconomic groups in the municipality.

## Conclusion

This study found a positive effect of physical fitness on post-compulsory education commencement by following pupils over a two-year period after the completion of compulsory education. Physical fitness showed strong evidence of an indirect effect on post-compulsory education commencement through the mediator academic achievement. A direct effect (i.e., not involving academic achievement) could not be established, but the width of the confidence intervals prevents firm conclusions on this part.

## Abbreviations

CI: 95% confidence interval
ECTS: European Credit Transfer and Accumulation System
ISCED: International Standard Classification of Education
OR: Odds ratio
