# Correction to: The indirect and direct pathways between physical fitness and academic achievement on commencement in post-compulsory education in a historical cohort of Danish school youth

**DOI:** 10.1186/s12889-017-4775-9

**Published:** 2017-09-25

**Authors:** Mikkel Porsborg Andersen, Liis Starkopf, Maurizio Sessa, Rikke Nørmark Mortensen, Henrik Vardinghus-Nielsen, Henrik Bøggild, Theis Lange, Christian Torp-Pedersen

**Affiliations:** 1 Public Health and Epidemiology Group, Department of Health Science and Technology, Aalborg University, Niels Jernes Vej 12 Øst, 9220 Aalborg, Denmark; 2 Section of Biostatistics, Department of Public Health, Faculty of Health and Medical Sciences, University of Copenhagen, Øster Farimagsgade 5, 1353 København K, Denmark; 3 Department of Experimental Medicine, Section of Pharmacology “L. Donatelli”, Second University of Naples, Via De Crecchio 7, 80138 Naples, Italy; 4 Department of Clinical Epidemiology, Aalborg University Hospital, Sdr. Skovvej 15, 9000 Aalborg, Denmark; 5 Center for Statistical Science, Peking University, Beijing, China

## Erratum

After publication of the article [1], it has been brought to our attention that one of the authors had their name spelt incorrectly. The second author should be “Liis Starkopf”. This has been corrected in the original version of the article.
